# Scanned: The global investments in computer-aided detection and ultraportable X-ray for tuberculosis

**DOI:** 10.1371/journal.pgph.0004232

**Published:** 2025-03-17

**Authors:** Juno Min, Jarred Halton, Catherine Villegas, Arlene Chua, Catherine Hewison, Stijn Deborggraeve

**Affiliations:** 1 Médecins Sans Frontières, International, Amsterdam, The Netherlands; 2 Médecins Sans Frontières, International, Geneva, Switzerland; 3 Médecins Sans Frontières, Operational Centre Brussels, Brussels, Belgium; 4 Médecins Sans Frontières, Operational Centre, Paris, France; 5 Médecins Sans Frontières, International, Brussels, Belgium

Chest X-ray (CXR) is an essential tool for screening and early detection of pulmonary tuberculosis (TB). CXR is more sensitive than symptom screening alone, frequently facilitating the detection of TB prior to the onset of symptoms [1].

Computer-aided detection (CAD) is artificial intelligence-based technology that the World Health Organization (WHO) has endorsed for CXR interpretation for TB screening and triage in individuals aged 15 years and older. CAD can facilitate the interpretation of CXR images in settings where there is limited availability of expert readers, such as radiologists. In general, CAD products provide a score as output (e.g., 0-1, 0-100), representing the likelihood of TB based on CXR findings. The immediate availability of results can increase workflow efficiency, decrease turnaround time and increase the number of individuals screened. Additional benefits include supporting non-experts in CXR interpretation and reducing reliance on human resources, such as radiologists. CAD also increases the cost-efficiency of TB screening by optimizing selection of individuals for confirmatory testing and decreasing the use of diagnostic consumables (e.g., GeneXpert cartridges) [2–4].

**Fig 1:**
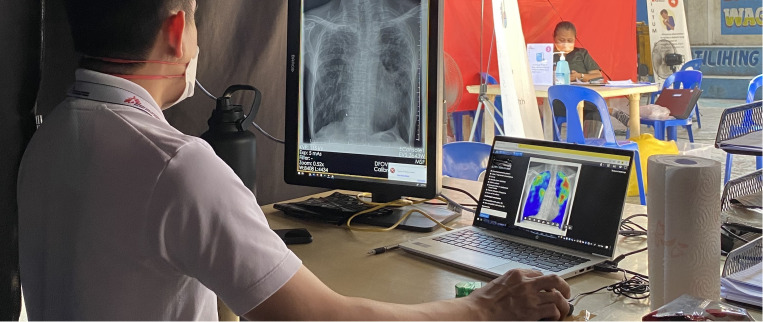
MSF physician using computer-aided detection (CAD) software to support chest X-ray interpretation during TB active case finding in Manila, Philippines.

Médecins Sans Frontières (MSF) first implemented CAD in 2022, in a TB project in the Philippines, where digital CXR with CAD was integrated into active case finding activities in a densely populated urban district (Tondo) of Manila (Fig 1). This implementation has demonstrated to us the direct benefits and significant impact of CAD in this setting. Since October 2022, CAD has helped screen over 20,000 individuals in Tondo district (on average, 100 per day) and nearly 5% of all screened individuals have been diagnosed with microbiologically confirmed TB. More than 60% of the confirmed TB cases did not report symptoms of TB and would have been missed by symptom screening alone, emphasizing the important role of CXR and CAD in the screening process.

Our experiences have also highlighted the challenges of CAD implementation with digital CXR. The WHO based their 2021 recommendation on analysis of 3 CAD software packages: CAD4TB (Delft Imaging), Lunit Insight CXR (Lunit Insight) and qXR (Qure.ai), but implementors may not be aware of the differences in performance between the many CAD solutions currently available (>20) and more guidance is needed to support user selection of a CAD system. An updated WHO policy statement on the use of CAD and a list of CAD solutions that passed an expert assessment of performance is expected in 2025, which will help inform implementors. Understanding both the limitations and risks of CAD is also essential for its effective use. CAD for TB detection is currently not recommended by WHO for use in individuals under 15 years of age and has decreased accuracy in those with a prior history of TB and in people living with HIV [1]. Some CAD products claim to detect radiographic findings beyond just TB, such as nodules, consolidation, and pleural effusion, but there is limited evidence to support these claims [2]. Additionally, because overreliance on CAD is a potential risk, it must be implemented carefully with adequate training and follow-up. Furthermore, in our experience, CAD struggles to verify the quality of a CXR image and we have seen that poor quality images can result in unreliable CAD scores. In general, we believe there should be increased awareness of these limitations and improved guidance and training for implementors. We have seen CAD systems unused or incorrectly used in countries because implementation protocols and follow-up support were not available.

CAD threshold selection is crucial and remains a significant challenge. During CAD implementation, a threshold score must be selected to refer individuals with a CAD score above it for confirmatory testing (e.g., with WHO recommended molecular tests such as GeneXpert or Truenat). However, CAD scores vary across contexts due to factors such as TB prevalence, co-morbidities in the population and X-ray equipment used. Therefore, it is essential to calibrate the threshold to local conditions. Additionally, CAD scores differ between providers, and even between software versions of the same CAD product. When selecting the threshold, it is also important to account for programmatic goals and capacity for confirmatory testing. There remains a notable lack of guidance on CAD threshold selection. Although the WHO Special Programme for Research and Training in Tropical Diseases (TDR) has published a toolkit to support the local calibration of CAD thresholds [5], implementation requires significant time and resources which may not be feasible for some projects. There is very limited information about alternative options for threshold selection and many users are unaware that the threshold can be changed and should be locally calibrated. In the absence of adequate instruction, implementers may default to a generic threshold or rely on a threshold suggested by the manufacturer which may not be appropriate for the local context. This not only leads to inefficient use of critical resources but can also result in missed TB diagnoses and highlights the urgent need for specific and practical protocols for threshold selection.

Ultraportable X-ray is an important innovation that has increased access to X-ray services, broadening the use of CXR beyond traditional hospital and clinic settings. This enables TB screening activities in diverse locations, including remote and hard-to-reach areas. These ultraportable X-ray devices are compact (e.g., able to be hand-carried or transported by vehicle), are versatile in set-up, operate on battery and do not require onsite services and maintenance, as the equipment can be transported to central facilities for repair [6]. However, there are significant trade-offs: image quality is decreased compared to standard fixed X-ray systems, patient positioning is more difficult, the lifespan of the equipment is shorter, and use is generally limited to the chest and extremities. Unfortunately, the limitations of ultraportable devices are often underestimated when selecting X-ray equipment, particularly in low- and middle-income countries where the availability of X-ray at hospital-level is limited [7]. With a total price of up to $100,000 for an ultraportable X-ray device, accessories, CAD and a 3-year warranty extension, investments costs are very high [8] and can represent an inefficient use of resources if these X-ray devices are not correctly and optimally utilized. Market shaping efforts are required to lower the total costs associated with the procurement and operation of digital X-ray systems including the hardware, software, maintenance, replacement of parts, training, re-training and disposal of equipment. This would be helpful not just for TB screening, but for a broader set of conditions which require imaging at the primary care level [9].

There is a pressing need for development of standards for ultraportable X-ray machines and their safe operation. In a rapidly expanding market of ultraportable X-ray equipment, many systems are being sold that do not meet the technical specifications developed by WHO for safety and image quality [10]. More pragmatic guidance, taking into account national regulatory processes, for ultraportable machines is required. For instance, in some countries these machines are purchased but are restricted to use within dedicated lead–lined or similarly protected X-ray departments, limiting their intended portability. Furthermore, there needs to be increased awareness about the appropriate use of ultraportable machines, which are often marketed as the “easier” option without adequate information on their specific applications. Ultraportable units should not replace fixed X-ray systems in hospitals but should ideally be used where fixed systems are impractical and portability is essential, such as for TB screening in remote areas or distant communities without local access to X-ray.

The innovations of CAD and ultraportable X-ray, frequently used together, have triggered significant promotion and investment by donors and international agencies to implement these technologies in high TB burden countries. While these tools are highly valuable in the appropriate contexts, we perceive significant shortcomings that must be addressed to ensure optimal utilization by national TB programs and implementers. While it is encouraging that CAD and ultraportable X-ray machines are receiving substantial funding for national TB programs, it is imperative for donors to acknowledge the importance of practical implementation and the necessity for comprehensive technical training for users. Equally crucial is addressing the complexities of procurement beyond initial purchase, such as managing and transporting spare parts, covering licensing fees, extending warranties, and responsibly disposing of old equipment. Additionally, securing funding for follow-up molecular diagnostic tests and treatment is essential to get the most value out of investments in X-ray and CAD.
